# Therapeutic efficacy of combined active and passive immunization in ART-suppressed, SHIV-infected rhesus macaques

**DOI:** 10.1038/s41467-022-31196-5

**Published:** 2022-06-16

**Authors:** Victoria E. K. Walker-Sperling, Noe B. Mercado, Abishek Chandrashekar, Erica N. Borducchi, Jinyan Liu, Joseph P. Nkolola, Mark Lewis, Jeffrey P. Murry, Yunling Yang, Romas Geleziunas, Merlin L. Robb, Nelson L. Michael, Maria G. Pau, Frank Wegmann, Hanneke Schuitemaker, Emily J. Fray, Mithra R. Kumar, Janet D. Siliciano, Robert F. Siliciano, Dan H. Barouch

**Affiliations:** 1 Center for Virology and Vaccine Research, Beth Israel Deaconess Medical Center, Boston, MA USA; 2 Bioqual, Rockville, MD USA; 3 Gilead Sciences, Foster City, CA USA; 4 US Military HIV Research Program, Walter Reed Army Institute of Research, Silver Spring, MD 20910 USA; 5 Janssen Vaccines & Prevention, Leiden, The Netherlands; 6 Department of Medicine, Johns Hopkins University School of Medicine, Baltimore, MD USA; 7 Ragon Institute of MGH, MIT, and Harvard, Cambridge, MA USA

**Keywords:** Translational immunology, Immunotherapy, Viral reservoirs, HIV infections

## Abstract

The latent viral reservoir is the critical barrier for developing an HIV-1 cure. Previous studies have shown that therapeutic vaccination or broadly neutralizing antibody (bNAb) administration, together with a Toll-like receptor 7 (TLR7) agonist, enhanced virologic control or delayed viral rebound, respectively, following discontinuation of antiretroviral therapy (ART) in SIV- or SHIV-infected rhesus macaques. Here we show that the combination of active and passive immunization with vesatolimod may lead to higher rates of post-ART virologic control compared to either approach alone. Therapeutic Ad26/MVA vaccination and PGT121 administration together with TLR7 stimulation with vesatolimod resulted in 70% post-ART virologic control in SHIV-SF162P3-infected rhesus macaques. These data suggest the potential of combining active and passive immunization targeting different immunologic mechanisms as an HIV-1 cure strategy.

## Abstract (web summary)

Antiretroviral therapy alone is insufficient in curing HIV-1 infection, due to latent viral reservoir persistency. Here, authors explore the post-virologic control of combining active and passive immunisation with vesatolimod, in a SHIV-infected rhesus macaque model.

## Introduction

Antiretroviral therapy (ART) alone does not cure HIV-1 infection due to the persistence of the latent viral reservoir^1^. Therapeutic vaccination is one of the strategies being pursued for functional cure and seeks to induce immunological control in the absence of ART^2^. The “shock and kill” method seeks to reactivate virus in the latent reservoir while virologically suppressed on ART followed by eliminating infected cells by an additional pharmacological or immunological intervention^2^. Previous work in SHIV-infected macaques has shown that administration of the V3-specific bNAb PGT121 together with a toll-like receptor 7 (TLR7) agonist delayed viral rebound following ART discontinuation^3^. We have also previously shown that Ad26/MVA vaccination together with TLR7 agonist administration resulted in improved virologic control and lower setpoint viral loads in SIV-infected macaques following ART discontinuation^4^. A human trial assessing Ad26 and MVA vaccination strategies demonstrated safety and immunogenicity in humans as well as a trend towards delayed rebound following ART interruption^5^. In the present study, we evaluate the therapeutic potential of combining active and passive immunologic strategies in ART-suppressed, SHIV-infected rhesus macaques. We assessed therapeutic efficacy of Ad26/MVA vaccination, PGT121 administration, and the TLR7 agonist vesatolimod in ART-suppressed, SHIV-SF162P3-infected rhesus macaques that initiated ART during early acute infection.

## Results

### Study design

**Fig. 1:**
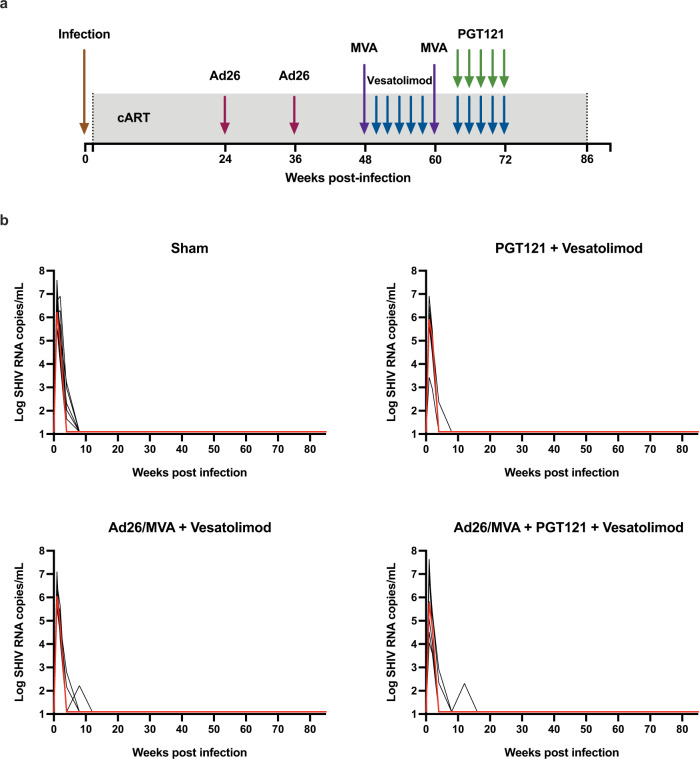
SHIV plasma virus became fully suppressed in all macaques during ART administration. **a** Study schematic. **b** SHIV viral loads from infection to cessation of ART with the median viral load of each group indicated by a red line.

We infected 51 Indian-origin Rhesus macaques (*Macaca mulatta*) intrarectally with 500 TCID_50_ SHIV-SF162P3 challenge stock and initiated ART on day 9 following infection with a preformulated ART cocktail (tenofovir disoproxil fumarate, emtricitabine, dolutegravir)^4^ (Fig. 1a). The peak median plasma viral loads for each treatment group prior to ART were 5.78–6.23 log RNA copies/mL (full range: 3.43–7.64; Fig. 1b; Supplementary Fig. 1), and plasma viremia decreased to undetectable levels between 4 and 16 weeks after initiation of ART (Fig. 1b).

Daily ART was administered for 85 weeks. Animals then received sham (group 1; *n* = 15), PGT121 (group 2; *n* = 12), Ad26/MVA vaccines (group 3; *n* = 12), or PGT121 and Ad26/MVA vaccines (group 4; *n* = 12, although *n* = 10 evaluable animals as 2 were euthanized prior to vaccination for reasons unrelated to the treatment interventions). Monkeys in groups 2–4 also received ten oral administrations of 0.15 mg/kg vesatolimod (Gilead Sciences) every 2 weeks from weeks 50 to 58 and from weeks 64 to 72. Groups 2 and 4 received intravenous infusions of 10 mg/kg PGT121 (Catalent Biopharma) every 2 weeks from weeks 64 to 72. Groups 3 and 4 were vaccinated intramuscularly with a total of 3 × 10^10^ viral particles (vp) of Ad26 vectors^5,6^ expressing SIV_smE543_ *gag-pol*, HIV-1 mosaic-1 *env*, and HIV-1 mosaic-2 *env*, at weeks 24 and 36 and boosted with 10^8^ plaque-forming units (pfu) MVA^5^ expressing SIV_smE543_ *gag-pol*, HIV-1 mosaic-1 *env-gag-pol*, and HIV-1 mosaic-2 *env-gag-pol* at weeks 48 and 60. ART was continued for an additional 14 weeks for all animals following the final dose of PGT121 to allow for the antibody to wash out. Viral loads remained suppressed with no evidence of viral blips from week 16 to week 86, at which point ART was discontinued (Fig. 1b).

### Pharmacokinetics and pharmacodynamics of vesatolimod and PGT121

We have previously reported induction of innate immune activation and robust CD4 T-cell activation in rhesus macaques treated with TLR7 agonists GS-986 or vesatolimod^3,4^. In this study, we similarly observed cellular immune activation on day 1 after treatment with vesatolimod. CD69 expression on CD4 T, CD8 T, and natural killer (NK) cells increased in the animals treated with vesatolimod (Supplementary Fig. 2; CD4s: *p* < 0.01; CD8s, NKs: *p* < 0.0001). Consistent with these findings, serum cytokines Eotaxin (*p* < 0.0001), I-TAC (*p* < 0.0001), IL-1RA (*p* < 0.0001), IL-8 (*p* < 0.0001), and MCP-1 (*p* < 0.0001) were induced in the animals treated with vesatolimod but not in sham-treated animals^3^ (Supplementary Fig. 3).

In the animals treated with PGT121, antibody pharmacokinetics were measured in serum from the day after the first infusion in week 64 through week 72 with minimal evidence of induction of anti-drug antibodies (Extended Data Figs. 3, 4). Only one animal in the groups treated with PGT121 developed anti-drug antibodies prior to the final infusion, although 6 of 22 animals treated with PGT121 eventually developed anti-drug antibodies after the PGT121 infusions were complete (Supplementary Fig. 5). Given the short half-life of the antibody, PGT121 became undetectable (<0.5 μg/mL) in the sera of all but one animal by 13 weeks after the final infusion (Supplementary Fig. 4).

### Immunogenicity of Ad26/MVA vaccines

**Fig. 2:**
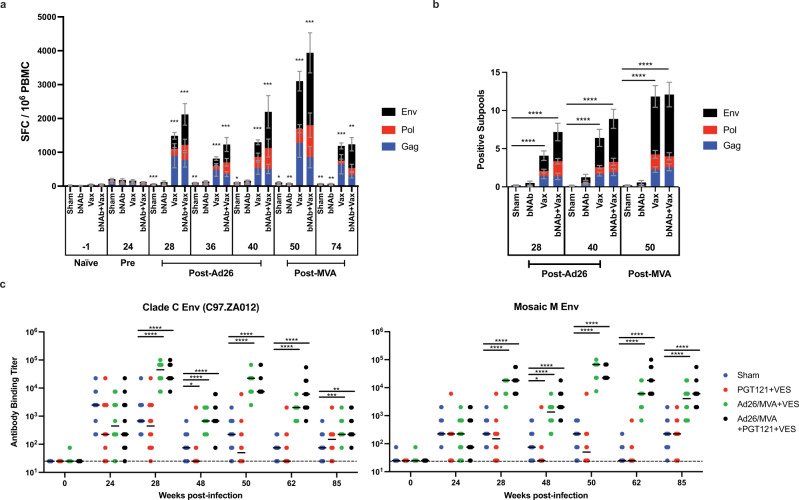
Induction of immune responses to treatment interventions. **a** Vaccination significantly and durably increased the size of SHIV-specific cellular immune IFN-γ responses over time in comparisons with pre-vaccination responses (“Pre”, week 24). Mean ± standard error is shown. **b** Vaccination significantly increased the breadth of SHIV-specific cellular immune IFN-γ responses in comparison to sham treatment. Means and standard errors are shown. **c** Vaccination significantly increased antibody binding to clade C and mosaic (mosaic 1) HIV-1 Env with median indicated. Two-sided Mann–Whitney tests were used to determine significance in non-paired analyses (**a**, **c**), and two-sided Wilcoxon matched-pairs sign rank tests were used in paired analyses (**b**). Sham = sham treatment; ‘bNAb’ or ‘PGT121 + VES’ = PGT121 + vesatolimod treatment; ‘Vax’ or ‘Ad26/MVA + VES’ = Ad26/MVA + vesatolimod treatment; and ‘bNAb + Vax’ or ‘Ad26/MVA + PGT121 + VES’ = Ad26/MVA, PGT121, + vesatolimod treatment. *p < 0.05 (in **a**, from left to right, *p* = 0.184 and 0.0339 for Sham; in **c**, *p* = 0.0465 in Clade C and 0.0489 in Mosaic M), **p < 0.01 (in **a**, from left to right*, p* = 0.0072 and 0.0016 for Sham, 0.0024 and 0.0068 for bNAb, and 0.002 for bNAb + Vax; in **c**, *p* = 0.0013), ***p < 0.001 (in **a**, from left to right, *p* = 0.005 for Sham, 0.0005 for all Vax, and 0.001 for bNAb + Vax from weeks 28 to 50; in **c**, *p* = 0.0008), ****p < 0.0001.

Animals that received the Ad26/MVA vaccines developed robust Gag-, Pol-, and Env-specific cellular immune responses following vaccination compared to pre-vaccination responses as measured by IFN-γ ELISPOT assays (Fig. 2a). All animals showed low responses at week 24 as a result of several weeks of SHIV-SF162P3 replication following infection. These responses increased in vaccinated animals at week 28 following the initial Ad26 priming, and further increased at week 50 following the initial MVA boosting. The unvaccinated animals trended towards decreasing responses during this time period (Fig. 2a).

Ad26/MVA vaccination also markedly increased the cellular immune breadth to Env, Gag, and Pol as compared to the unvaccinated groups (Fig. 2b). Responses to subpools of peptides spanning HIV-1 PTE Env (*n* = 49), SIV_mac239_ Pol (*n* = 26), and SIV_mac239_ Gag (*n* = 13) measured via IFN-γ ELISPOT assays were used to estimate the breadth of responses. In both groups of vaccinated animals, the number of subpool responses to Gag, Pol, and Env epitopes was greater than in the unvaccinated animals at weeks 28, 40, and 50 (*p* < 0.0001, all comparisons). Cellular immune breadth was greatest at week 50 where there was an overall average of 12.0 subpool responses per animal with an average 7.8 responses for Env, 1.7 for Pol, and 2.4 for Gag. The limited availability of cells precluded fine epitope mapping, and thus the actual breadth of T-cell responses in these animals may be higher.

Vaccination also induced Env-specific antibody responses by ELISA compared to the unvaccinated animals (Fig. 2c). Responses to the HIV-1 clade C Env C97.ZA012 were comparable in all groups at week 24 prior to vaccination. Following vaccination, antibody titers increased by 1.5–1.8 log at weeks 28 and 50. Similar increases were observed in vaccinated animals to the HIV-1 group M mosaic (mosaic 1) Env.

### Proviral DNA prior to ART interruption

Intact cell-associated SHIV DNA was detectable in approximately one-third of macaques at weeks 82–85. We isolated DNA from magnetically purified, PBMC-derived CD4 T cells and measured the number of intact proviruses using the SHIV-specific version of the intact proviral DNA assay (IPDA)^7^. Due to limited availability of PBMCs, the average lower limit of detection was 31.9 copies per million CD4 T cells (Supplementary Fig. 6). The average number of detectable intact proviruses in sham controls was borderline at 63.5 copies per million CD4 T cells (range: 24.8–205.1), likely reflecting the early initiation of ART on day 9 and the 85 weeks of continuous ART. As 22 out of 49 animals had intact proviral frequencies below the limit of detection, we were unable to compare differences among groups. The small size of the viral reservoirs in these infected animals that were treated with ART during acute infection contrasts with substantially larger viral reservoirs in infected animals that were treated with ART during chronic infection (average 249.6 intact proviruses per million CD4 T cells).

### Viral rebound following ART discontinuation

**Fig. 3:**
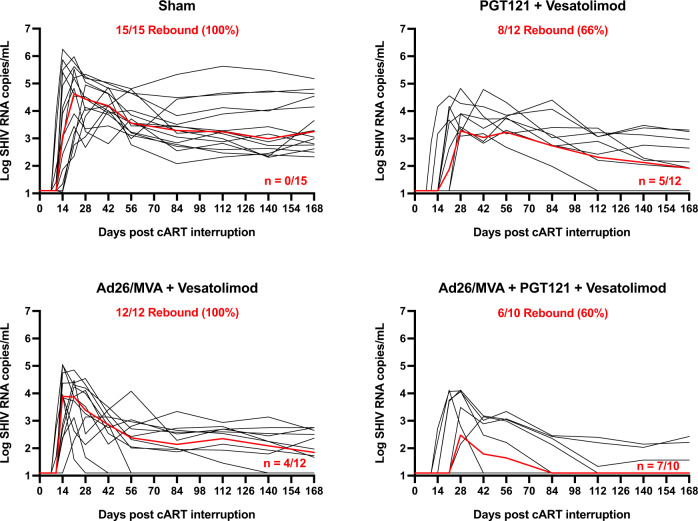
The combination of vesatolimod, Ad26/MVA, and PGT121 treatment resulted in the fewest viremic animals post-ART cessation. Plasma SHIV viral loads from the cessation of ART to day 168 with the median viral load of each group indicated by a red line. The number and percentage of animals in each group that rebounded are indicated below the group label, and the number of animals in the group with a negative viral load at the end of the surveillance period is indicated in the lower right corner of each graph.

At week 86, ART was discontinued in all macaques, and plasma viral loads were monitored for 168 days following ART discontinuation to assess viral rebound (Fig. 3). In the sham control group, 15 of 15 macaques (100%) rebounded within 3 weeks with a high peak and setpoint viral loads typical of SHIV-SF162P3 infection^3,8,9^. In the group that received PGT121 and vesatolimod, 8 of 12 macaques (66%) rebounded within 168 days and one subsequently developed virological control. In the group that received Ad26/MVA vaccines and vesatolimod, 12 of 12 animals (100%) rebounded and 4 animals developed post-rebound virological control. In the group that received PGT121, Ad26/MVA vaccines, and vesatolimod, only 6 of 10 macaques (60%) rebounded and 3 animals showed post-rebound virological control. Thus, by day 168 following ART discontinuation, 7 of 10 macaques (70%) in the combined group had undetectable viral loads as compared with 0 of 15 macaques (0%) in the sham control group.

**Fig. 4:**
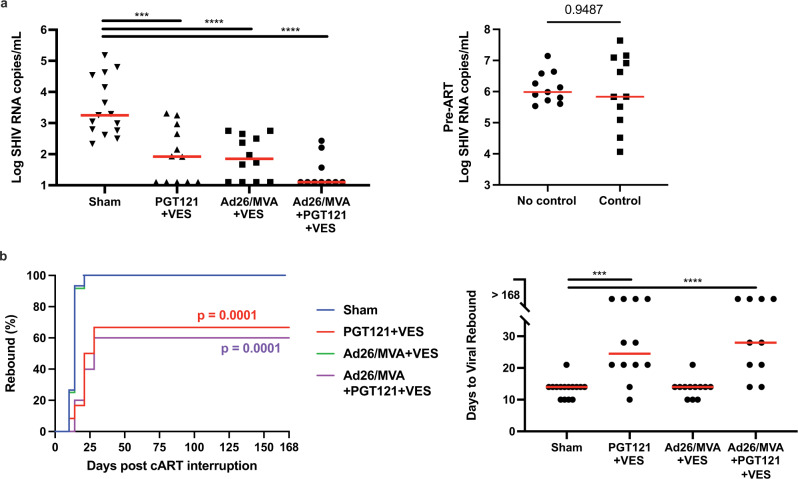
All treatment interventions reduced post-treatment interruption viral loads. **a** Treatment interventions induced significantly lower setpoint viral loads as compared to sham (left). There was no association of virologic control with pre-ART peak viral loads in the Ad26/MVA vaccinated animals (right). Two-sided Mann–Whitney tests used to determine significance. **b** PGT121 treatment significantly delayed viral rebound. The Kaplan–Meier plot shows the significant difference between the PGT121 groups and sham group, determined via the Mantel-Cox log-rank test (PGT121, *χ*^2^ = 14.37, *p* = 0.0001; Ad26/MVA + PGT121, *χ*^2^ = 14.85, *p* = 0.0001). The dot-plot visualizes the days to rebound (median indicated in red). Two-sided Mann–Whitney tests used to determine significance. Sham = sham treatment; PGT121 + VES = PGT121 + vesatolimod treatment; Ad26/MVA + VES = Ad26/MVA + vesatolimod treatment; and Ad26/MVA + PGT121 + VES = Ad26/MVA, PGT121, + vesatolimod treatment. Horizontal red lines indicate median values. ***p < 0.001 (in **a**, *p* = 0.0008; in **b**, *p* = 0.0002), ****p < 0.0001.

All three groups that received immunologic interventions demonstrated reduced setpoint viral loads compared to the sham controls. Sham controls showed median setpoint viral loads of 3.5 log copies/mL, and the combined group showed the largest reduction of median setpoint viral loads to 1.1 log copies/mL (*p* < 0.0001; Fig. 4a). The PGT121 + vesatolimod group had median setpoint viral loads of 1.9 log copies/mL (*p* = 0.0008), and the Ad26/MVA + vesatolimod group had median setpoint viral loads of 1.9 log copies/mL (*p* < 0.0001).

The PGT121 + vesatolimod and PGT121 + Ad26/MVA + vesatolimod groups demonstrated substantially delayed rebound (a median 24.5 and 28 days, respectively) in comparison to the sham (median 14 days; *p* = 0.0002, *p* < 0.0001, respectively; Fig. 4b). Moreover, 4 animals in each of these groups did not rebound during the follow-up period. The animals that did not rebound did not show higher PGT121 concentrations or lower pre-ART peak viral loads^3^ (Supplementary Fig. 7). Both groups that received PGT121 with vesatolimod exhibited delayed viral rebound compared with sham controls.

The breadth of cellular immune responses at week 50 to each pool of Env, Pol, and Gag epitopes correlated with setpoint viral loads following ART interruption (Spearman’s *r* = −0.409 to −0.455; *p* = 0.001–0.0035; Supplementary Fig. 8a). The magnitude of ELISPOT responses (SFC per million PBMCs) at week 50 also correlated with setpoint viral loads (Spearman’s *r* = −0.363 to −0.448; *p* = 0.0013–0.0086; Supplementary Fig. 8b). The combined Env+Gag+Pol breadth (Spearman’s *r* = −0.482; *p* = 0.0005) and magnitude (Spearman’s *r* = −0.483; *p* = 0.0004) correlated best with setpoint viral loads (Supplementary Fig. 8a, b). Binding antibody titers against both the HIV-1 Clade C (Spearman’s *r* = −0.287; *p* = 0.0453) and group M mosaic (Spearman’s *r* = −0.382; *p* = 0.0067) Env also weakly correlated with setpoint viral loads (Supplementary Fig. 8c).

Taken together, these data suggest that the combination of PGT121, Ad26/MVA, and vesatolimod led to both post-rebound virologic control, which was comparable with the Ad26/MVA + vesatolimod group, and delayed viral rebound, which was comparable with the PGT121 + vesatolimod group. Thus, combination of active and passive immunization with vesatolimod appeared to provide additive benefits of both interventions.

## Discussion

Our data demonstrate that the combination of PGT121, Ad26/MVA vaccines, and vesatolimod led to 70% virologic control following ART discontinuation in SHIV-SF162P3-infected macaques that initiated ART during acute infection. In the triple combination group, 4 of 10 animals did not rebound during the follow-up period, and 3 additional animals that did rebound progressed to post-rebound virologic control. This study shows the proof-of-concept that combining active and passive immunization, together with TLR7 stimulation, leads to therapeutic efficacy that phenotypically and quantitatively appears to be additive for the two modalities. Combining multiple immunologic mechanisms may be a promising approach to improving reservoir targeting and achieving virologic control following ART discontinuation.

PGT121 with vesatolimod may target the reservoir and delay or even prevent viral rebound by targeting provirus-containing cells during ART suppression^3^. While PGT121 has been shown to induce direct antiviral activity in macaques previously^10^, we do not believe that direct antiviral activity of PGT121 explains the delayed rebound over 168 days following ART discontinuation, since PGT121 levels were undetectable at the time of ART interruption. We were unable to prove a direct effect on the latent reservoir, however, because the frequency of intact proviruses as measured by IPDA was largely at or below the detection limit in all groups including the sham controls due to early initiation of ART and the duration of treatment. However, in our previous study combining PGT121 and vesatolimod^3^, in which ART was started on day 7 of infection, we demonstrated that adoptive transfer and CD8 T-cell depletion failed to produce any evidence of residual infectious virus. Future studies will explore these interventions in animals that initiated ART during chronic infection.

Ad26/MVA vaccination with vesatolimod did not appear to target the reservoir directly but robustly increased Env/Gag/Pol-specific cellular immune responses, which likely contributed to virologic control following ART discontinuation, as we showed previously with Ad26/MVA vaccines and the related TLR7 agonist GS-986 in SIV-infected macaques^4^. Median setpoint viral loads were lower in vaccinated animals compared with sham controls, and approximately one-third of animals in each of the vaccine groups that rebounded then showed post-rebound virologic control to undetectable levels. Most clinical trials of HIV-1 cure strategies require re-initiation of ART shortly after viral rebound, and thus this phenomenon of post-rebound virologic control may be missed in clinical trials as currently designed.

Our results combining Ad26/MVA vaccination and PGT121 administration in the presence of TLR7 stimulation show that combining immunological interventions has the potential to improve post-ART virologic control. While we have shown that combining bNAb therapy with TLR7 stimulation during ART delays or prevents viral rebound in macaques starting ART 1 week post-infection^3^, Hsu et al. have found that macaques treated with ART at 2 weeks post-infection followed by bNAbs PGT121 and N6-LS and TLR7 agonist administration had a more modest effect^11^, potentially due to the strong anti-drug antibody responses to N6-LS. An Ad26/Env vaccine recently showed no significant efficacy as a preventative HIV-1 vaccine in the HVTN705 study (NCT03060629).

Future studies could explore the potential utility of additional latency reactivating agents (LRAs). The second mitochondria-derived activator of caspases (SMAC) mimetic AZD5582 has been used an LRA in the rhesus macaque model to induce transient SIV viremia via noncanonical NF-kB signaling^12^. However, the combination of the SMAC mimetic and bispecific HIVxCD3 DART molecules was unable to induce transient viremia or decrease the size of the latent reservoir in SHIV-infected rhesus macaques that began ART in chronic infection^13^ likely because an anti-drug antibody response was raised against the bispecific antibodies^14^. Treatment with bNAbs in lieu of ART very early in infection has previously been shown to induce virologic control in slightly more than half of SHIV-infected macaques through a CD8+ T-cell-dependent mechanism^15,16^, indicating a treatment strategy incorporating early immunologic interventions may also be of use in combination with later treatments. Optimizing the combinations of immunologic interventions such as LRAs, bNAbs, and vaccines could be evaluated for increased therapeutic efficacy and more effective delay, prevention, or control of viral rebound following ART discontinuation.

In summary, our data demonstrate the proof-of-concept that combining active and passive immunization can achieve additive therapeutic effects in ART-suppressed SHIV-infected rhesus macaques, resulting in a high rate of virologic control upon discontinuation of ART. Given the limitations of macaque models, clinical trials combining this regimen of bNAbs, therapeutic vaccination, and TLR7 agonist are planned in HIV-1-infected humans.

## Methods

### Animals

All animal studies were approved by the appropriate Institutional Animal Care and Use Committee (IACUC) at Beth Israel Deaconess Medical Center and Bioqual. Fifty-one outbred, Indian-origin young adult rhesus macaques (*Macaca mulatta*) were genotyped for the protective MHC class I alleles Mamu-A*01, Mamu-B*08, and Mamu-B*17 and susceptible and resistant TRIM5α alleles. Animals were otherwise randomly allocated to the four groups. The study was not powered for comparisons across intervention groups. All monkeys were housed at Bioqual (Rockville, MD). Animals were infected intrarectally with a single 500 TCID_50_ dose of our SHIV-SF162P3 challenge stock, and ART was initiated on day 9. SHIV-SF62P3 (GenBank accession number KF042063) is derived from SIV_mac239_, with the *tat*, *rev*, and *env* of HIV-1_SF162_ replacing the analogous SIV regions^17^. Monkeys were bled up to twice per week for viral load measurements, and immunological and virological assays were performed blinded.

### ART regimen

The preformulated combined antiretroviral therapy (ART) cocktail (Gilead) contained 5.1 mg/mL tenofovir disoproxil fumarate (TDF), 40 mg/mL emtricitabine (FTC), and 2.5 mg/mL dolutegravir (DTG) in a solvent containing 15% (v/v) kleptose adjusted to pH 4.2. ART cocktail was subcutaneously administered once daily at 1 mL/kg body weight.

### Treatment interventions

Macaques were divided into the following treatment groups: sham (group 1; *n* = 15), PGT121 (group 2; *n* = 12), Ad26/MVA vaccines (group 3; *n* = 12), or PGT121 and Ad26/MVA vaccines (group 4; *n* = 12, but *n* = 10 evaluable animals as 2 were euthanized prior to vaccination for anesthetic complications unrelated to treatment interventions). Monkeys in groups 2–4 also received ten oral administrations of 0.15 mg/kg vesatolimod (Gilead Sciences) every 2 weeks from weeks 50 to 58 and from weeks 64 to 72. Groups 2 and 4 received intravenous infusions of 10 mg/kg PGT121 (Catalent Biopharma) every 2 weeks from weeks 64 to 72. Groups 3 and 4 were vaccinated intramuscularly with a total of 3 × 10^10^ viral particles (vp) of Ad26 vectors^5,6^ expressing SIV_smE543_ *gag-pol*, HIV-1 mosaic-1 *env*, and HIV-1 mosaic-2 *env*, at weeks 24 and 36 and boosted with 10^8^ plaque-forming units (pfu) MVA^5^ expressing SIV_smE543_ *gag-pol*, HIV-1 mosaic-1 *env-gag-pol*, and HIV-1 mosaic-2 *env-gag-pol* at weeks 48 and 60 (Supplementary Fig. 9). ART was continued for an additional 14 weeks for all animals following the final dose of PGT121 to allow for the antibody to wash out.

### PGT121 pharmacokinetics and anti-drug antibody (ADA) assays

The pharmacokinetics of PGT121 in the macaques were assayed as previously described^3^. Briefly, ELISAs were performed with plates coated with clade C (C97ZA.012) gp140 Env protein capture reagent before being washed with PBS (0.05% Tween 20) and blocked with Blocker Casein (Pierce). Samples were incubated on the plates for an hour prior to washing and incubation with a mouse PGT121 anti-idiotype monoclonal antibody at 1 μg/mL. After another wash and incubation with rabbit anti-mouse IgG horseradish peroxidase (Thermo Scientific), plates were washed and developed for 5 min with SureBlue (KPL Laboratories) and stopped with TMB Stop Solution (KPL Laboratories). Plates were read on a VersaMax microplate reader (Molecular Devices) at 450 nm with SoftmaxPro v6.5.1. ADA assays were performed using the MesoScale Discovery (MSD) electrochemiluminescence (ECL) platform also as previously described^3^. Briefly, serum samples diluted in assay buffer were incubated overnight at 4 °C on an orbital plate shaker with sulfo-tagged and biotinylated PGT121 (0.5 μg/mL each). After incubating the mixtures on a streptavidin-functionalized plate, the plates were washed prior to the addition of tripropylamine (TPA). Luminescence was read using the MSD SQ-120 ECL imager and was proportional to the amount of ADA. The 95th percentile of ECL signal response of naïve macaque serum was used as the cut-off point for assay positivity.

### Cellular immune assays

HIV- and SIV-specific cellular immune responses were assessed by IFN-γ ELISPOT assays essentially as described^3^. Estimates of cellular immune breadth involved IFN-γ ELISPOT assays using subpools of peptides across the SIV_mac239_ Gag (*n* = 13), SIV_mac239_ Pol (*n* = 26), and HIV-1 Env (*n* = 49) proteins. Flow cytometric staining was performed with predetermined titres of monoclonal antibodies at concentrations suggested by the manufacturer (Becton Dickinson unless noted) against CD3 (SP34; Alexa Fluor 700), CD4 (OKT4; BV510, Biolegend), CD8 (SK1; APC-Cy7), CD14 (M5E2; BUV737), CD16 (3G8; BV650), CD25 (PE-Cy7; M-A251), CD28 (L293; PerCP-Cy5.5), CD38 (APC; HB-7), CD56 (NCAM16; BV786), CD69 (TP1.55.3; PE-TexasRed; Beckman Coulter), CD95 (DX2; BV711), CCR5 (3A9; PE), CCR7 (3D12; BV421), HLA-DR (BUV-395; G46-6), Ki67 (B56; FITC), and PD-1 (EH21.1; BV605).

### Plasma cytokine analysis

The ProcartaPlex multiplex immunoassay (Thermo Fisher) was used to determine EDTA plasma cytokine levels according to the manufacturer’s instructions for 15 cytokines (Eotaxin, IFN-a, IFN-g, IL-1B, IL-1RA, IL-12p40, IL-2, IL-6, IL-8, IL-10, IP-10, I-TAC, MCP-1, MIG, and TNF-a). Samples were read on a Luminex 200 platform and analyzed using Bio-plex Manager v6.2 software (Bio-Rad).

### Viral RNA assays

Viral RNA extracted from plasma with the QIAmp Viral RNA Kit (Qiagen) was converted to cDNA with Superscript III VILO (Invitrogen), and the SHIV cDNA was quantified via qPCR with QuantStudio 1.7.1 as previously described^18^ using the following primers and probe with TaqMan Fast Advanced Master Mix (Applied Biosystems): Fwd, 5′-GTCTGCGTCATCTGGTGCATTC-3′; Rev, 5′-CACTAGGTGTCTCTGCACTATCTGTTTTG-3′; and Probe 5′-(FAM) CTTCCTCAGTGTGTTTCACTTTCTCTTCTGCG-(BHQ)-3′.

### Intact proviral DNA assay (IPDA)

CD4+ T cells were isolated from frozen PBMCs with the EasySep Non-Human Primate CD4+ T Cell Isolation Kit (Stem Cell), and total cellular DNA was extracted with the QIAmp DNA Blood Mini Kit (Qiagen). Absolute quantification of intact proviral SHIV DNA was performed using a SHIV-specific version of previously described via digital droplet PCR assays^7,19^ with 300 ng of input DNA, and cell equivalents were determined by quantifying copies of macaque RPP30 in parallel (average of 31.8 × 10^3^ ± standard deviation 0.7 × 10^3^ cell equivalents per well). Detail of the SHIV IPDA will be described elsewhere (Fray et al., in preparation).

### Statistical analyses

Virological and immunological data analysis was performed using GraphPad Prism Version 9.3.0 (GraphPad Software). Two-sided Mann–Whitney tests and Wilcoxon signed-rank tests were used to compare groups. Two-sided Spearman rank-correlation tests were conducted for correlation analyses. Sample sizes were not predetermined via statistical methods, and, except where stated, the experiments were not randomized nor were investigators blinded to the allocation of samples during the experiments and outcome assessment.

### Reporting summary

Further information on research design is available in the Nature Research Reporting Summary linked to this article.

## Supplementary information

Supplementary Information

Reporting Summary

## Data Availability

Raw data for individual monkeys is shown in the figures. All relevant data generated and analyzed in this study are available with the article in the source data. Any additional data are available from the corresponding author upon reasonable request. Source data are provided with this paper.

## Source data

Source Data
