# Supplementary material for: Therapeutic efficacy of combined active and passive immunization in ART-suppressed, SHIV-infected rhesus macaques
Source: Nat Commun. 2022 Jun 16;13:3463. doi: 10.1038/s41467-022-31196-5 (PMC9203527; doi:10.1038/s41467-022-31196-5)
Supplement: Supplementary file 1 — Supplementary Information [file 41467_2022_31196_MOESM1_ESM.pdf]

**Supplementary Material for:**

# Therapeutic efficacy of combined active and passive immunization in ART-suppressed, SHIV-infected rhesus macaques

Victoria E.K. Walker-Sperling[1], Noe B. Mercado[1], Abishek Chandrashekar[1], Erica N. Borducchi[1], Jinyan Liu[1], Joseph P. Nkolola[1], Mark Lewis[2], Jeffrey P. Murry[3], Yunling Yang[3], Romas Geleziunas[3], Merlin L. Robb[4], Nelson L. Michael[4], Maria G. Pau[5], Frank Wegmann[5], Hanneke Schuitemaker[5], Emily J. Fray[6], Mithra R. Kumar[6], Janet D. Siliciano[6], Robert F. Siliciano[6], Dan H. Barouch[1,7*]

[1]Center for Virology and Vaccine Research, Beth Israel Deaconess Medical Center, Boston, MA, USA.

[2]Bioqual, Rockville, MD, USA.

[3]Gilead Sciences, Foster City, CA, USA.

[4]US Military HIV Research Program, Walter Reed Army Institute of Research, Silver Spring, Maryland 20910, USA.

[5]Janssen Vaccines & Prevention, Leiden, Netherlands.

[6]Department of Medicine, Johns Hopkins University School of Medicine, Baltimore, MD, USA.

[7]Ragon Institute of MGH, MIT, and Harvard, Cambridge, MA, USA.

*Corresponding author: D.H.B. (dbarouch@bidmc.harvard.edu)

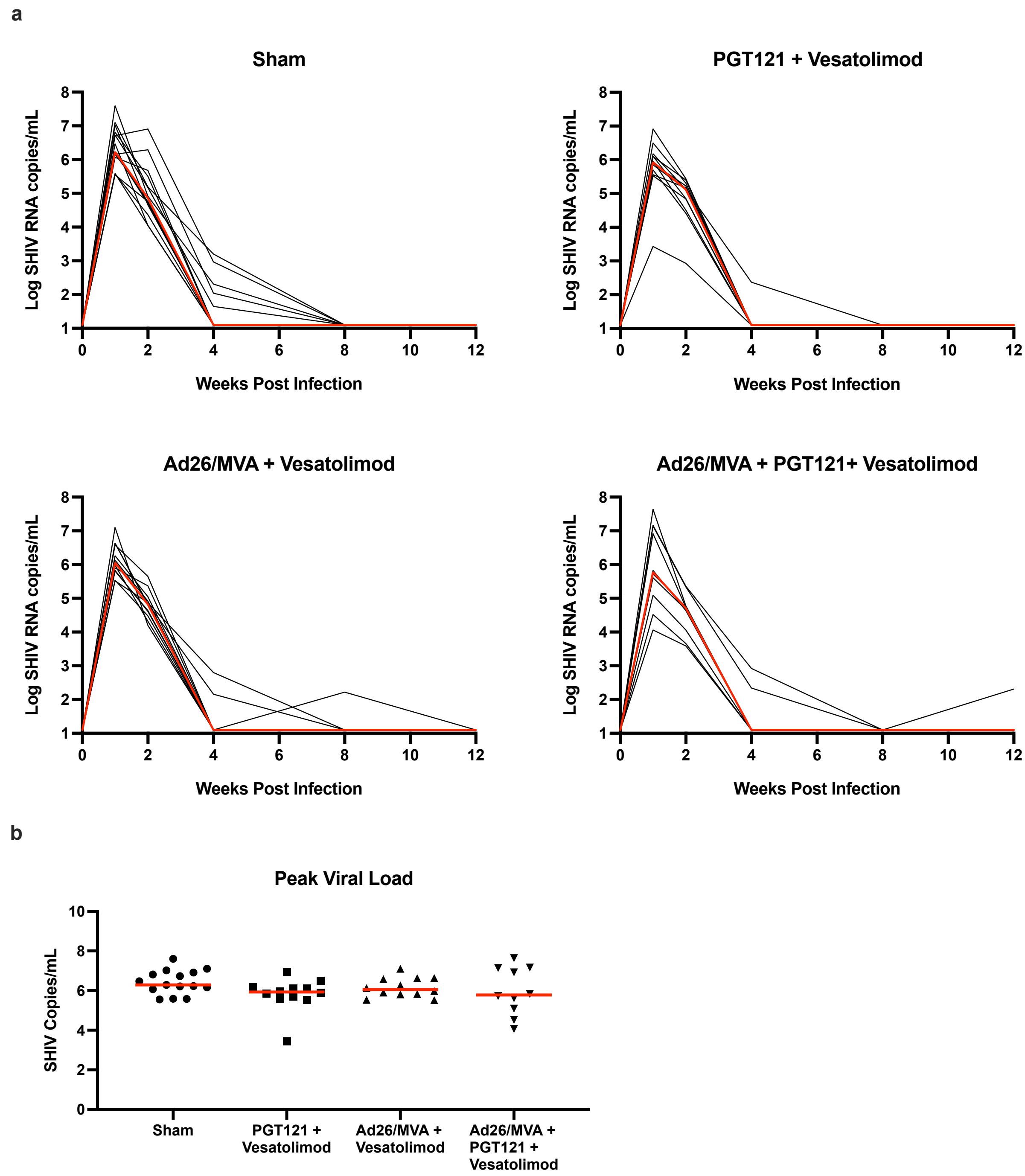


**Supplementary Fig. 1. SHIV plasma viremia early in infection. a,** SHIV viral loads from infection to week 10 of infection with the median viral load of each group indicated by a red line. **b**, Peak viral loads with the median viral load indicated in red. N = 51 macaques; $n_{Sham}$ = 15

macaques, $n_{PGT121+Vesatolimod}$ = 12 macaques, $n_{Ad26/MVA+Vesatolimod}$ = 12 macaques, and $n_{Ad26/MVA+PGT121+Vesatolimod}$ = 10 macaques.

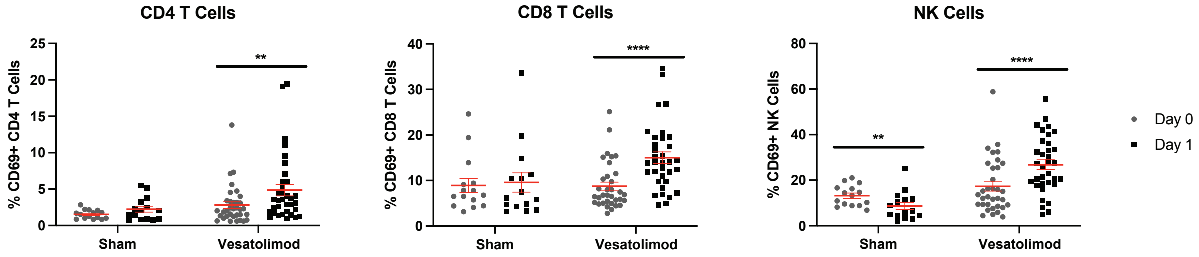


**Supplementary Fig. 2. Vesatolimod induces cellular activation in vivo.** Bulk PBMCs were analyzed via flow cytometry to determine the effect of the TLR7 agonist on activation in T and NK cells. CD69 expression was found to significantly increase in CD4 and CD8 T cells and NK cells as a result of TLR7 engagement. Representative data shown from week 64 on the day of but prior to injection with vesatolimod and the following day. Average ± standard error of the mean is shown. Two-sided Wilcoxon signed rank tests used to determine significance. N = 51 macaques; $n_{Sham}$ = 15 macaques, $n_{Vesatolimod}$ = 39 macaques. ** $p < 0.01$ ($p = 0.0023$ (CD4 T cells, Vesatolimod) and 0.0067 (NK cells, Sham)), **** $p < 0.0001$

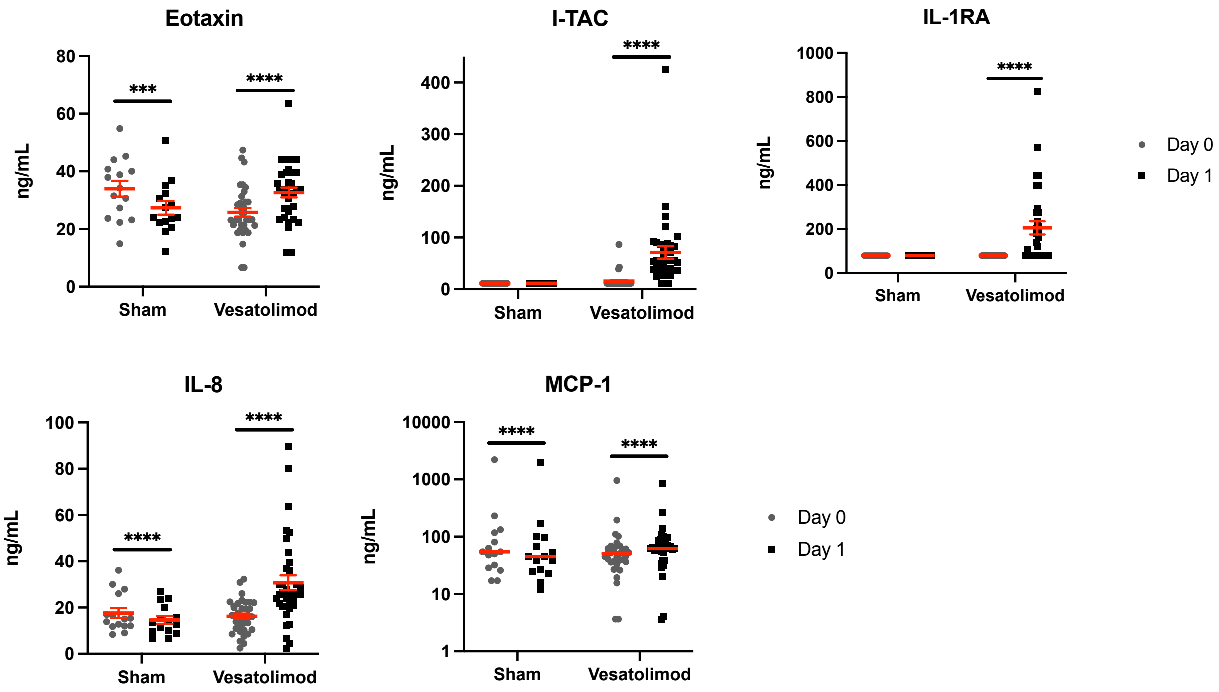


**Supplementary Fig. 3. Vesatolimod induces cytokine production in vivo.** Serum cytokines were measured before and after vesatolimod administration. A total of nine pairs of measurements were taken for each animal with the median values displayed for each animal. Average ± standard error of the mean shown save for with MCP-1, where the median is shown. Two-sided Wilcoxon signed rank tests used to determine significance. N = 51 macaques; $n_{Sham}$ = 15 macaques, $n_{Vesatolimod}$ = 39 macaques. *** $p < 0.001$ ($p = 0.0002$ in Eotaxin), **** $p < 0.0001$

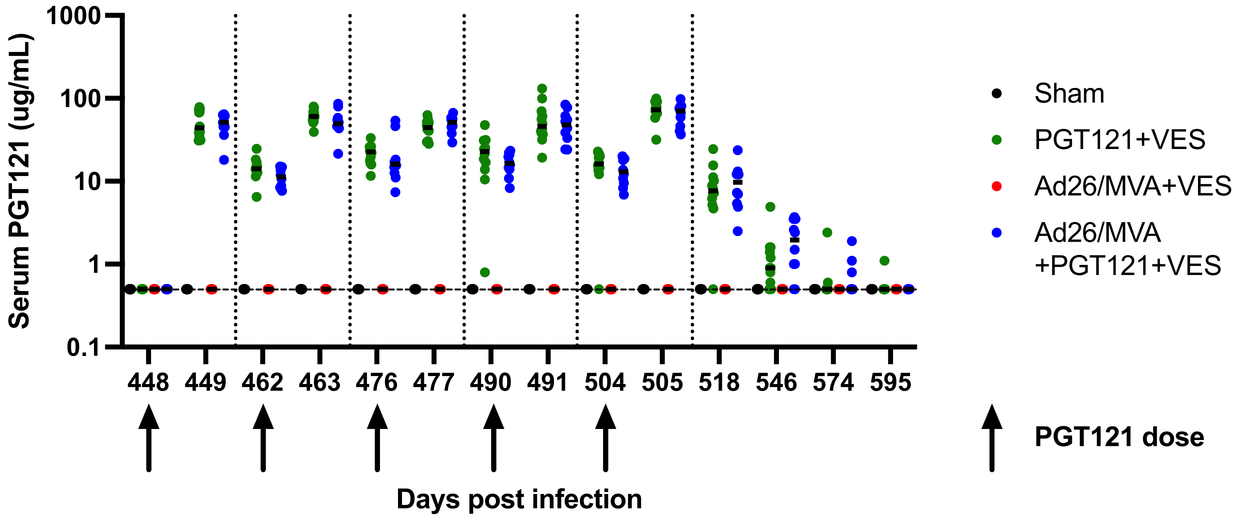


**Supplementary Fig. 4. PGT121 serum pharmacokinetics.** Serum concentration of PGT121 was measured by ELISA in all the animals from the first day of PGT121 treatment to one week prior to ART cessation. Arrows indicate the timing of PGT121 infusion. Sham = sham treatment; PGT121 + VES = PGT121 + vesatolimod treatment; Ad26/MVA + VES = Ad26/MVA + vesatolimod treatment; and Ad26/MVA + PGT121 + VES = Ad26/MVA, PGT121, + vesatolimod treatment. N = 51 macaques; $n_{Sham}$ = 15 macaques, $n_{PGT121+Vesatolimod}$ = 12 macaques, $n_{Ad26/MVA+Vesatolimod}$ = 12 macaques, and $n_{Ad26/MVA+PGT121+Vesatolimod}$ = 10 macaques.

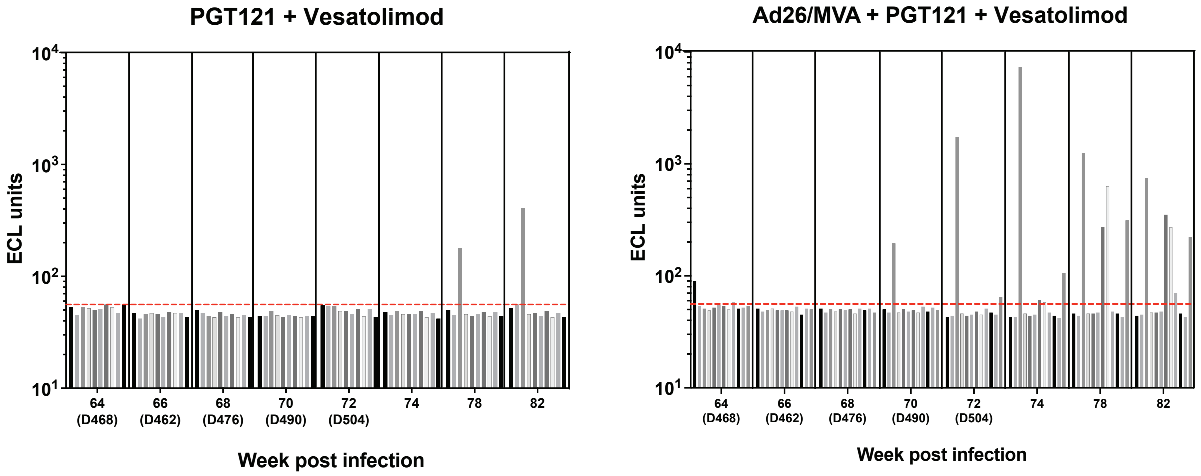


**Supplementary Fig. 5. PGT121 anti-drug antibody reactivity.** Serum reactivity of the treated animals to PGT121 was measured from the first administration of PGT121 to one month prior to the cessation of ART. ECL = electrochemiluminescence.

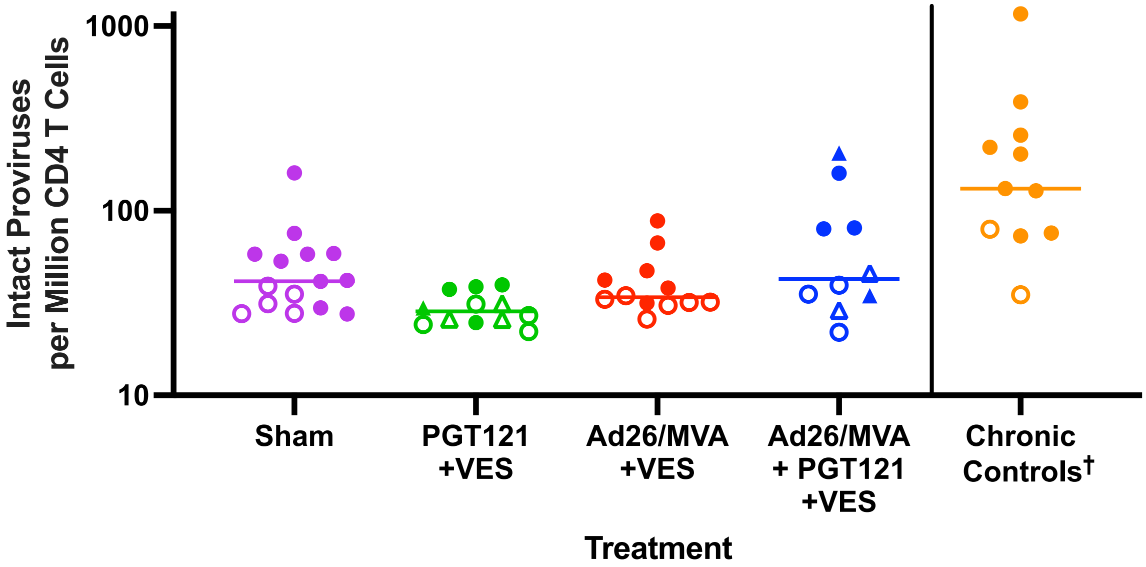


**Supplementary Fig. 6. Intact proviral frequency of SHIV in circulating CD4 T cells.** DNA extracted from peripheral blood CD4 T cells from week 82-85 post-ART initiation was analyzed for the frequency of intact provirus. All values were assayed with three replicate wells. Unfilled symbols represent the level of detection for animals in which the amount of virus was undetectable. Circles represent animals that rebounded after cessation of ART, and triangles represent animals that did not rebound for at least 168 days post cessation of ART. Lines indicate medians. †Reference values are from animals treated 58 weeks with ART following 89 weeks of chronic SHIV infection with the median value indicated. Sham = sham treatment; PGT121 + VES = PGT121 + vesatolimod treatment; Ad26/MVA + VES = Ad26/MVA + vesatolimod treatment; and Ad26/MVA + PGT121 + VES = Ad26/MVA, PGT121, + vesatolimod treatment. N = 51 macaques; $n_{\text{Sham}}$ = 15 macaques, $n_{\text{PGT121+Vesatolimod}}$ = 12 macaques, $n_{\text{Ad26/MVA+Vesatolimod}}$ = 12 macaques, and $n_{\text{Ad26/MVA+PGT121+Vesatolimod}}$ = 10 macaques.

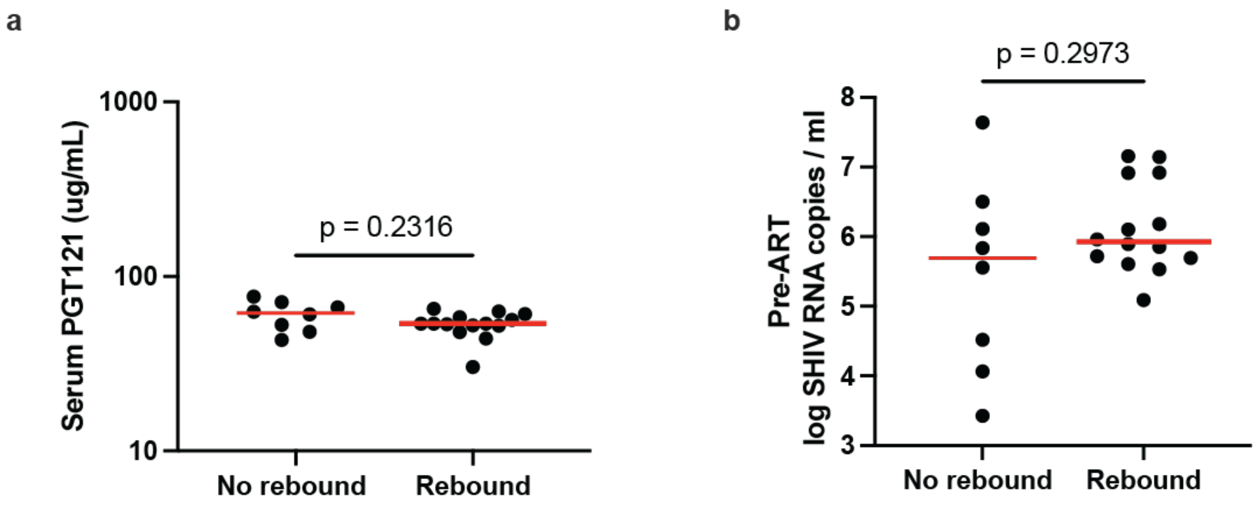


**Supplementary Fig. 7. No difference in median serum PGT121 concentration and pre-ART viral loads in animals that did and did not rebound. a,** Equivalent PGT121 concentrations in macaques that did and did not rebound**. b,** Equivalent pre-ART peak viral loads in macaques that did and did not rebound. Two-sided Mann-Whitney tests used to determine significance for both analyses. Median indicated by red bar. N = 22 macaques treated with PGT121 (PGT121 + vesatolimod and Ad26/MVA + PGT121 + vesatolimod groups); $n_{Rebound}$ = 8 macaques, $n_{No\ Rebound}$ = 14 macaques.

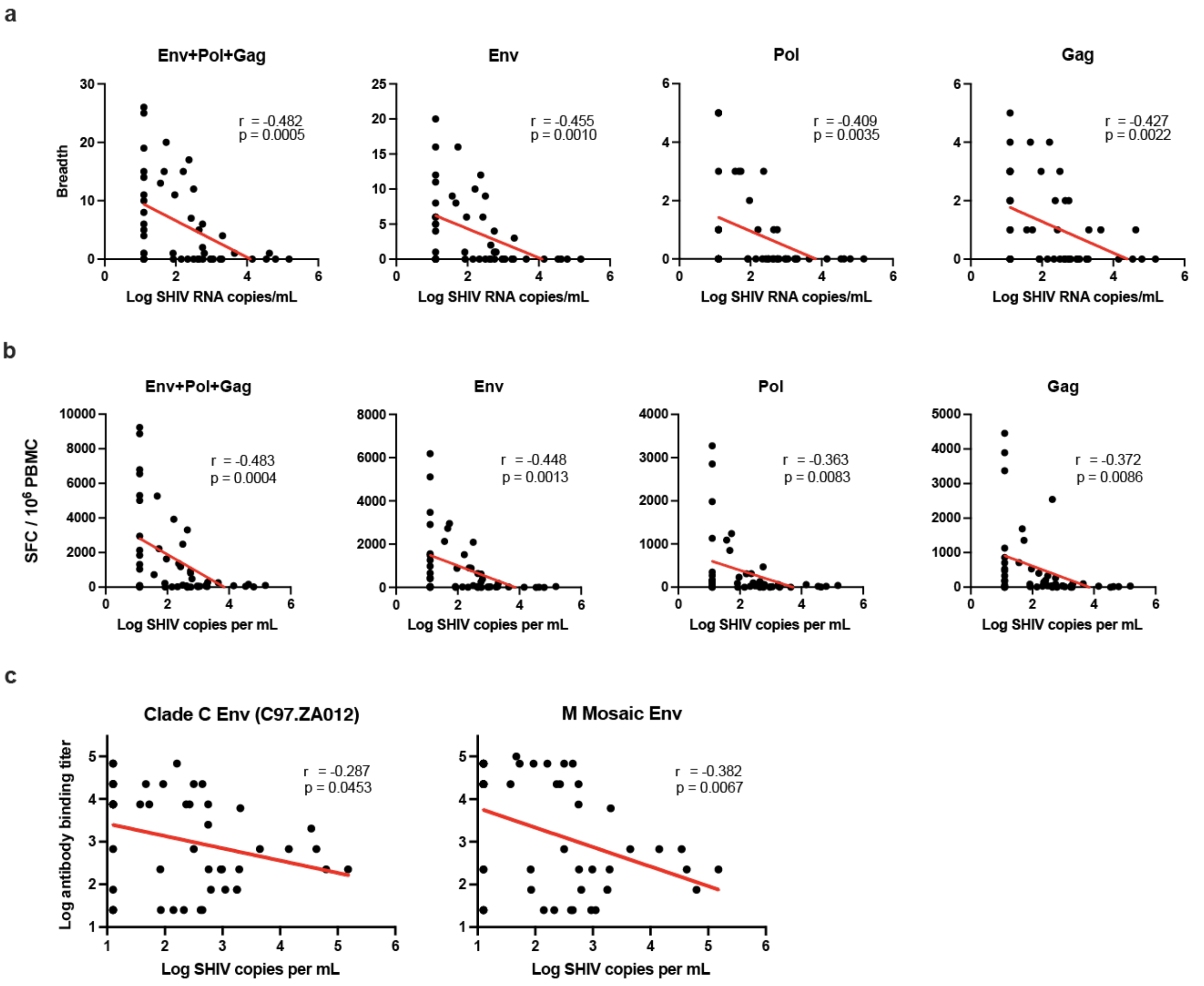


**Supplementary Fig. 8. Strength and breadth of adaptive immune responses correlate with improved SHIV outcomes. a,** Breadth of SHIV-specific cellular immune IFN-γ responses significantly correlate with set-point viral load post-ART cessation**. b,** Size of SHIV-specific cellular immune IFN-γ responses significantly correlate with set-point viral load post-ART cessation**. c,** Serum antibody binding to Env significantly correlates with set-point viral load post-ART cessation. Two-sided Spearman rank-correlation tests were used to determine significance. N = 51 macaques. * $p < 0.05$, ** $p < 0.01$, *** $p < 0.001$, **** $p < 0.0001$

```
                    10         20         30         40         50         60
HXB2        MRVK---EKY QHLWRWGWRW GTMLLGMLMI CSATEKLWVT VYYGVPVWKE ATTTLFCASD
SHIV-SF162  ....GIRKN. .....----G ..L....... ...V...... .......... ..........
Mosaic 1    ...TGIRKN. .....----. ......I... ...AG..... .......... ..........
Mosaic 2    ...RGIQRNW PQW.I----. .ILGFW.II. .RVMGN.... .......... .K........

                    70         80         90        100        110        120
HXB2        AKAYDTEVHN VWATHACVPT DPNPQEVVLV NVTENFNMWK NDMVEQMHED IISLWDQSLK
SHIV-SF162  .......... .......... ......I..E .......... .N........ ..........
Mosaic 1    .......... .......... .........E .......... .N........ ..........
Mosaic 2    ....EK.... .......... ......M..E .......... ....D..... ..R.......

                   130        140        150        160        170        180
HXB2        PCVKLTPLCV SLKC----TD LKNDTNTNSS SGRMIMEKGE IKNCSFNIST SIRGKVQKEY
SHIV-SF162  .......... T.H.----.N .E.A...TG. NWKE-.NR.. .......VT. ..GN.M....
Mosaic 1    .......... T.N.TDDVRN VT.NATNTN. .WGEP..... ........T. ...N....Q.
Mosaic 2    .......... T.E.----RN VR.VSSNGTY NIIHNETYK. M......AT. VVEDRK..VH

                   190        200        210        220        230        240
HXB2        AFFYKLDIIP ID-------- ND--TTSYKL TSCNTSVITQ ACPKVSFEPI PIHYCAPAGF
SHIV-SF162  .L.....VV. ..-------- ..--N.X.N. IN........ .......... ..........
Mosaic 1    .L.....VV. ..-------- ..SNN.N.R. I......... .......... ..........
Mosaic 2    .L..R...V. L.ENNSSEKS SENSSEY.R. IN....A... .......D.. .........Y

                   250        260        270        280        290        300
HXB2        AILKCNNKTF NGTGPCTNVS TVQCTHGIRP VVSTQLLLNG SLAEEEVVIR SVNFTDNAKT
SHIV-SF162  ......D.K. ..S...I... .......... .......... .....G.... .E.....V..
Mosaic 1    ......D.K. .......... .......... .......... .......... .E...N....
Mosaic 2    .......... ......N... ........K. .......... ......II.. .E.L.N....

                   310        320        330        340        350        360
HXB2        IIVQLNTSVE INCTRPNNNT RKRIRIQRGP GRAFVTIGK- IGNMRQAHCN ISRAKWNNTL
SHIV-SF162  .....KE... .......... ..S.P.--.. .K..YAT.DI ..DI...... ..GE......
Mosaic 1    .M....V... .......... ..S.H.--.. ....Y.A.DI ..DI...... ....N.....
Mosaic 2    ...H..ET.N .T........ ..S...--.. .QT.YAT.DI ..DI...... L..DG..K..

                   370        380        390        400        410        420
HXB2        KQIASKLREQ FGNNKTIIFK QSSGGDPEIV THSFNCGGEF FYCNSTQLFN STWF--NSTW
SHIV-SF162  ...VT..QA. .-E....V.. .......... M......... .........- ------....
Mosaic 1    R..VE..GK. .......V.N H......... M......... ......K... ...TWN....
Mosaic 2    QGVKK..A.H .-P....N.T S.....L..T ......R... ....TSG..- ------.G.Y

                   430        440        450        460        470        480
HXB2        STEGSNNTEG SDTITLPCRI KQIINMWQKV GKAMYAPPIS GQIRCSSNIT GLLLTRDGG-
SHIV-SF162  NNTIGP.-NT NG........ .....R..E. .........R .......... .........-
Mosaic 1    NNTKRS.-DT EEH....... ........E. .........R .......... .........-
Mosaic 2    MPN.T.S-NS .SN....... ........E. .R.......A .N.T.R.... .........S

                   490        500        510        520        530        540
HXB2        --NSNNESEI FRPGGGDMRD NWRSELYKYK VVKIEPLGVA PTKAKRRVVQ REKRAVGIGA
SHIV-SF162  -REVS.TT.. .......... .......... .......... .......... ......TL..
Mosaic 1    --.DTSGT.. .......... .......... .......... .......... S..S......
Mosaic 2    NNGVP.DT.T .........N .......... ..EVK..... ..E......E S..S......

                   550        560        570        580        590        600
HXB2        LFLGFLGAAG STMGAASMTL TVQARQLLSG IVQQQNNLLR AIEAQQHLLQ LTVWGIKQLQ
SHIV-SF162  V......... .......L.. .......... .......... .......... ..........
Mosaic 1    V......... .......... .....L.... .......... .......... ..........
Mosaic 2    V...I..... .......I.. .......... .....S.... .......M.. ..........

                   610        620        630        640        650        660
HXB2        ARILAVERYL KDQQLLGIWG CSGKLICTTA VPWNASWSNK SLEQIWNHTT WMEWDREINN
SHIV-SF162  ..V....... .......... .......... .......... ..D....NM. ....E...G.
Mosaic 1    ..V....... .......... .........T .......... ..DK...NM. ....E.....
Mosaic 2    T.V..I.... Q......L.. .......... ....T..... .QTD..DNM. ..Q..K..G.

                   670        680        690        700        710        720
HXB2        YTSLIHSLIE ESQNQQEKNE QELLELDKWA SLWNWFNITN WLWYIKLFIM IVGGLVGLRI
SHIV-SF162  ..N..YT... .......... .......... ......D.SK ......I... ..........
Mosaic 1    .....YT... .......... .......... ......D.S. ...------- ----------
Mosaic 2    ..GE.YR.L. .......... KD..A..S.K N.....D... ...------- ----------
```

```
                    730        740        750        760        770        780
HXB2        VFAVLSIVNR VRQGYSPLSF QTHLPTPRGP DRPEGIEEEG GERDRDRSIR LVNGSLALIW
SHIV-SF162  ..T....... .......... ..RF.A...L .......... ........RP ..H.L.....
Mosaic 1    ---------- ---------- ---------- ---------- ---------- ----------
Mosaic 2    ---------- ---------- ---------- ---------- ---------- ----------

                    790        800        810        820        830        840
HXB2        DDLRSLCLFS YHRLRDLLLI VTRIVELLGR RGWEALKYWW NLLQYWSQEL KNSAVSLLNA
SHIV-SF162  .......... .......I.. AA........ .........G ......I... .......FG.
Mosaic 1    ---------- ---------- ---------- ---------- ---------- ----------
Mosaic 2    ---------- ---------- ---------- ---------- ---------- ----------

                    850        860        870
HXB2        TAIAVAEGTD RVIEVVQGAC RAIRHIPRRI RQGLERILL
SHIV-SF162  I......... .I...A.RIG ..FL...... ......T..
Mosaic 1    ---------- ---------- ---------- ---------
Mosaic 2    ---------- ---------- ---------- ---------
```

**Supplementary Fig. 9. HIV Env protein alignment.** The HIV mosaic-1 and mosaic-2 and SHIV-SF162 Env proteins are shown aligned to the reference HXB2 sequence.
