# Supplementary material for: Therapeutic efficacy of combined active and passive immunization in ART-suppressed, SHIV-infected rhesus macaques
Source: Nat Commun. 2022 Jun 16;13:3463. doi: 10.1038/s41467-022-31196-5 (PMC9203527; doi:10.1038/s41467-022-31196-5)
Supplement: Supplementary file 2 — Reporting Summary [file 41467_2022_31196_MOESM2_ESM.pdf]

nature portfolio

Corresponding author(s): Dan H. Barouch

Last updated by author(s): May 20, 2022

Reporting Summary

Nature Portfolio wishes to improve the reproducibility of the work that we publish. This form provides structure for consistency and transparency in reporting. For further information on Nature Portfolio policies, see our Editorial Policies and the Editorial Policy Checklist.

Statistics

For all statistical analyses, confirm that the following items are present in the figure legend, table legend, main text, or Methods section.

n/a	Confirmed	
☐	☒	The exact sample size (*n*) for each experimental group/condition, given as a discrete number and unit of measurement
☐	☒	A statement on whether measurements were taken from distinct samples or whether the same sample was measured repeatedly
☐	☒	The statistical test(s) used AND whether they are one- or two-sided<br>*Only common tests should be described solely by name; describe more complex techniques in the Methods section.*
☐	☒	A description of all covariates tested
☐	☒	A description of any assumptions or corrections, such as tests of normality and adjustment for multiple comparisons
☐	☒	A full description of the statistical parameters including central tendency (e.g. means) or other basic estimates (e.g. regression coefficient) AND variation (e.g. standard deviation) or associated estimates of uncertainty (e.g. confidence intervals)
☐	☒	For null hypothesis testing, the test statistic (e.g. *F*, *t*, *r*) with confidence intervals, effect sizes, degrees of freedom and *P* value noted<br>*Give P values as exact values whenever suitable.*
☒	☐	For Bayesian analysis, information on the choice of priors and Markov chain Monte Carlo settings
☒	☐	For hierarchical and complex designs, identification of the appropriate level for tests and full reporting of outcomes
☒	☐	Estimates of effect sizes (e.g. Cohen's *d*, Pearson's *r*), indicating how they were calculated

Our web collection on statistics for biologists contains articles on many of the points above.

Software and code

Policy information about availability of computer code

Data collection	Softmax Pro version v6.5.1, QuantStudio 1.7.1
Data analysis	GraphPad Prism v9.3.0, Bio-plex Manager v6.2

For manuscripts utilizing custom algorithms or software that are central to the research but not yet described in published literature, software must be made available to editors and reviewers. We strongly encourage code deposition in a community repository (e.g. GitHub). See the Nature Portfolio guidelines for submitting code & software for further information.

Data

Policy information about availability of data

All manuscripts must include a data availability statement. This statement should provide the following information, where applicable:

- Accession codes, unique identifiers, or web links for publicly available datasets
- A description of any restrictions on data availability
- For clinical datasets or third party data, please ensure that the statement adheres to our policy

Raw data for individual monkeys is shown in the figures. All relevant data generated and analyzed in this study are available with the article in the source data. Any additional data are available from the corresponding author upon reasonable request.

Field-specific reporting

Please select the one below that is the best fit for your research. If you are not sure, read the appropriate sections before making your selection.

☒ Life sciences ☐ Behavioural & social sciences ☐ Ecological, evolutionary & environmental sciences

For a reference copy of the document with all sections, see nature.com/documents/nr-reporting-summary-flat.pdf

Life sciences study design

All studies must disclose on these points even when the disclosure is negative.

Sample size	No formal sample size calculation was performed. A sample size of N=51 (N=12/experimental group; N=15/sham) was selected based on our experience to date to achieve power to detect a 2-fold increase in time to viral rebound. Our previous studies Borducchi et al, 2016 (Nature, PMID: 27841870), and Borducchi et al, 2018 (Nature, PMID: 30283138), were able to detect significant differences with similar numbers of animals in each experimental group.
Data exclusions	No data were excluded.
Replication	All assays discussed in the manuscript were performed with at least two technical replicates, which were minimally different. As the details of the SHIV IPDA have not yet been published, the assay was run independently on all animals at least once in triplicate, and over half the samples were run an additional time to ensure consistency of results. All attempts at replication of all assays were successful, and multiple timepoints were assessed to ensure reproducibility. Three of the four arms of the macaque experiment itself have been previously published (Sham, PGT121 + Vesatolimod, and Ad26/MVA + another TLR7 agonist), and similar results in regards to post-treatment interruption rebound and virologic control for each group were obtained in this study as in those previous.
Randomization	Animals expressing protective MHC class I alleles and susceptible and resistant TRIM5 alleles were distributed among the groups. Animals were otherwise randomly allocated to groups.
Blinding	The animals were housed and treated at a separate facility from where sample processing and assays were performed, so all individuals involved with the latter performed the assays and data collection blinded. Group analyses were performed by unblinded researchers post-acquisition of data.

Reporting for specific materials, systems and methods

We require information from authors about some types of materials, experimental systems and methods used in many studies. Here, indicate whether each material, system or method listed is relevant to your study. If you are not sure if a list item applies to your research, read the appropriate section before selecting a response.

Materials & experimental systems

n/a	Involved in the study	
☐	☒	Antibodies
☒	☐	Eukaryotic cell lines
☒	☐	Palaeontology and archaeology
☐	☒	Animals and other organisms
☒	☐	Human research participants
☒	☐	Clinical data
☒	☐	Dual use research of concern

Methods

n/a	Involved in the study	
☒	☐	ChIP-seq
☒	☐	Flow cytometry
☒	☐	MRI-based neuroimaging

Antibodies

Antibodies used	Catalent: PGT121<br>Becton Dickenson: CD3 (SP34.2; Alexa Fluor 700), CD8 (SK1; APC-H7), CD14 (M5E2; BUV737), CD16 (3G8; BV650), CD25 (PE-Cy7; M-A251), CD28 (L293; PerCP-Cy5.5), CD38 (APC; HB-7), CD56 (NCAM16; BV786), CD95 (DX2; BV711), CCR5 (3A9; PE), CCR7 (3D12; BV421), HLA-DR (BUV-395; G46-6), Ki67 (B56; FITC), and PD-1 (EH21.1; BV605)<br>Biolegend: CD4 (OKT4; BV510)<br>Beckman Coulter: CD69 (TP1.55.3; PE-TexasRed)
Validation	PGT121 has previously been used as a therapeutic in other SHIV studies, including Borducchi et al, 2018 (Nature, PMID: 30283138). All other antibodies were used per manufacturer's instructions and validations on their websites and in line with previously published assays. We confirmed reactivity for rhesus monkey samples and titrated and tested the antibodies for specificity prior to use in all assays.

Animals and other organisms

Policy information about studies involving animals; ARRIVE guidelines recommended for reporting animal research

Laboratory animals	M. mulatta, mixed male and female, age 3-8 years.
Wild animals	The study did not involve wild animals.
Field-collected samples	The study did not involve samples collected from the field.
Ethics oversight	All animal studies were approved by the appropriate Institutional Animal Care and Use Committee (IACUC) at Beth Israel Deaconess Medical Center and Bioqual.

Note that full information on the approval of the study protocol must also be provided in the manuscript.
